# Supplementary material for: Diversity of cortical activity changes beyond depression during Spreading Depolarizations
Source: Nat Commun. 2023 Nov 25;14:7729. doi: 10.1038/s41467-023-43509-3 (PMC10676372; doi:10.1038/s41467-023-43509-3)
Supplement: Supplementary file 1 — Supplementary Information [file 41467_2023_43509_MOESM1_ESM.pdf]

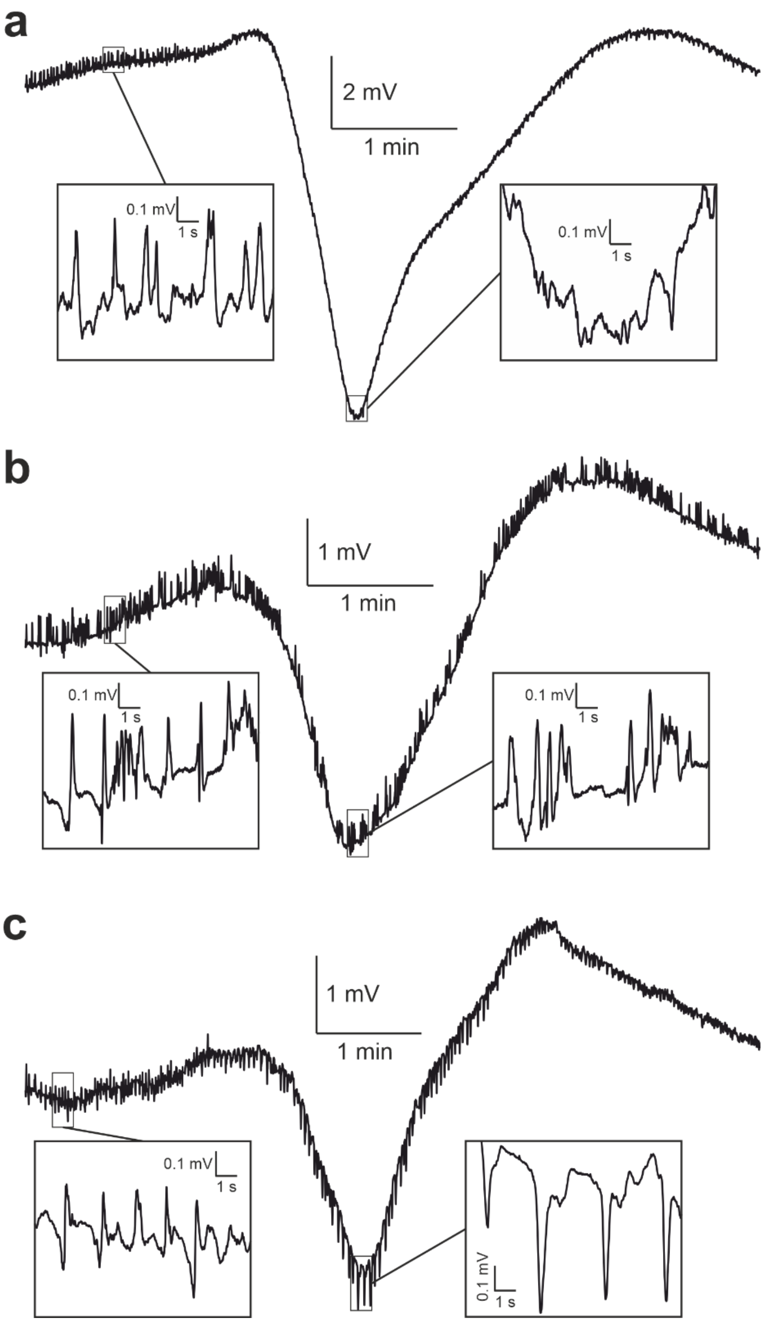

Supplementary Figure 1. Variable effects of spreading depolarization (SD) on electrocorticographic activity in human patients with subarachnoid hemorrhage. Three example SDs with DC-ECoG (full-band signal, from Fig. 1b) demonstrating: **a** depression, **b** no change and **c** boom on the ongoing cortical activity. The enlarged fragments show δ-activity during the episodes used as Pre-SD and SD for power ratio calculations in Fig. 1.

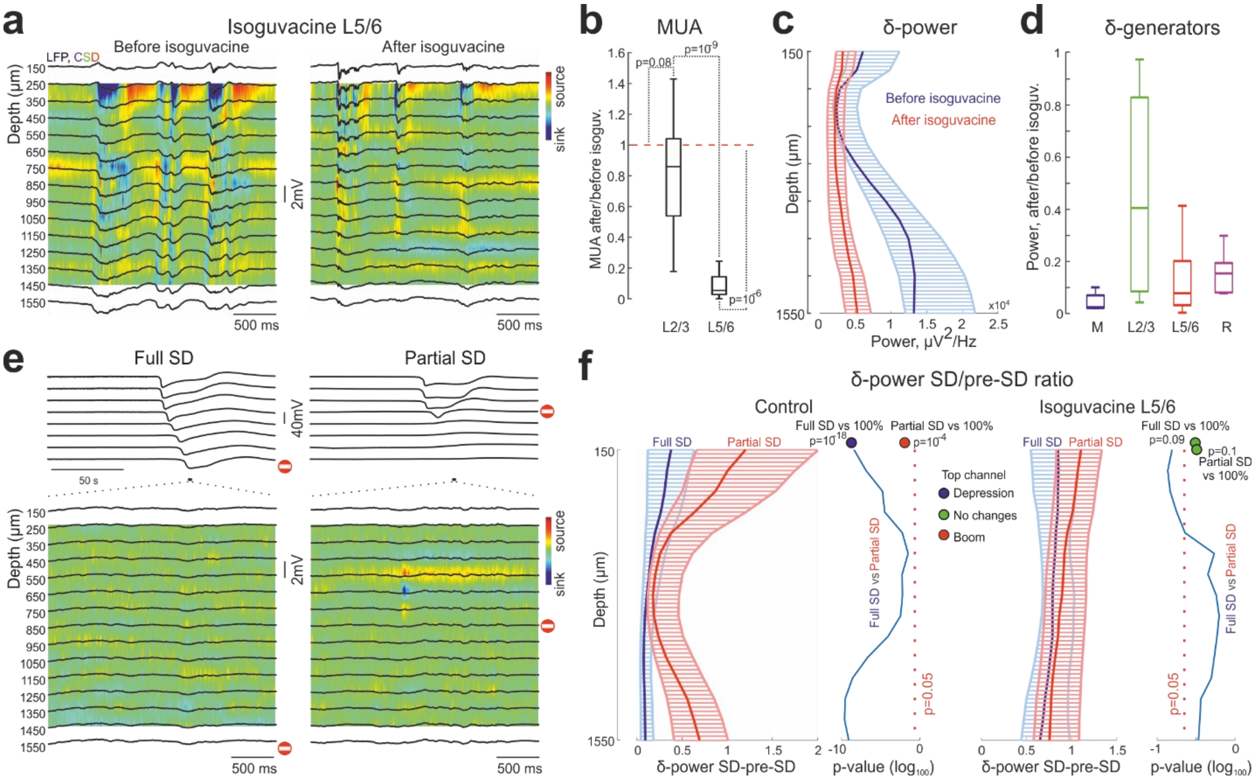

Supplementary Figure 2. Effects of inhibition of deep cortical layers on δ-activity during SDs. **a** Example traces of LFP (black) at different cortical depths overlaid on current-source density (CSD) map in before (left) after (right) isoguvacine injection at depth of 1200μm. **b** MUA frequency in superficial (L2/3) and deep (L5-6) layers after injection of the GABA(A) receptor agonist isoguvacine into the deep layers, normalized to the control MUA levels (Wilcoxon signed rank test (after vs before isoguvacine injection), Wilcoxon rank sum test (L2/3 vs L5/6), n=[25 (L2/3), 30 (L5/6)]. **c** Depth profile of LFP δ-power before (blue) and after (red) isoguvacine injection into deep cortical layers (median with 25th and 75th percentiles). **d** δ-generators power after injection of isoguvacine to deep layers normalized to the control values. b-d: Grouped data from 5 rats. **e** Example traces of FP at different cortical depths overlaid on CSD map during full SD (left) and partial SD terminated at depth of 850 μm (right). **f** Depth profile of SD/pre-SD ratio of LFP δ-power (median with 25th and 75th percentiles) for full SDs terminated at depth ≥ 1.2mm (blue) and superficial SDs terminated at depth ≤ 1mm (red), and corresponding p-values for a difference between full and superficial SDs in control conditions (left) and after isoguvacine injection into deep cortical layers (right). Circles above show p-values for a significance in difference of LFP δ-power at the top electrodes between SD and pre-SD epochs. Pooled data from: Control, 127 full SDs and 63 superficial SDs from 13 rats; Isoguvacine treatment, 25 full SDs and 53 superficial SDs from 5 rats (Wilcoxon rank sum test (full SDs vs partial SDs), Wilcoxon signed-rank test (SD/pre-SD ratio)). Source data are provided as a Source Data file.

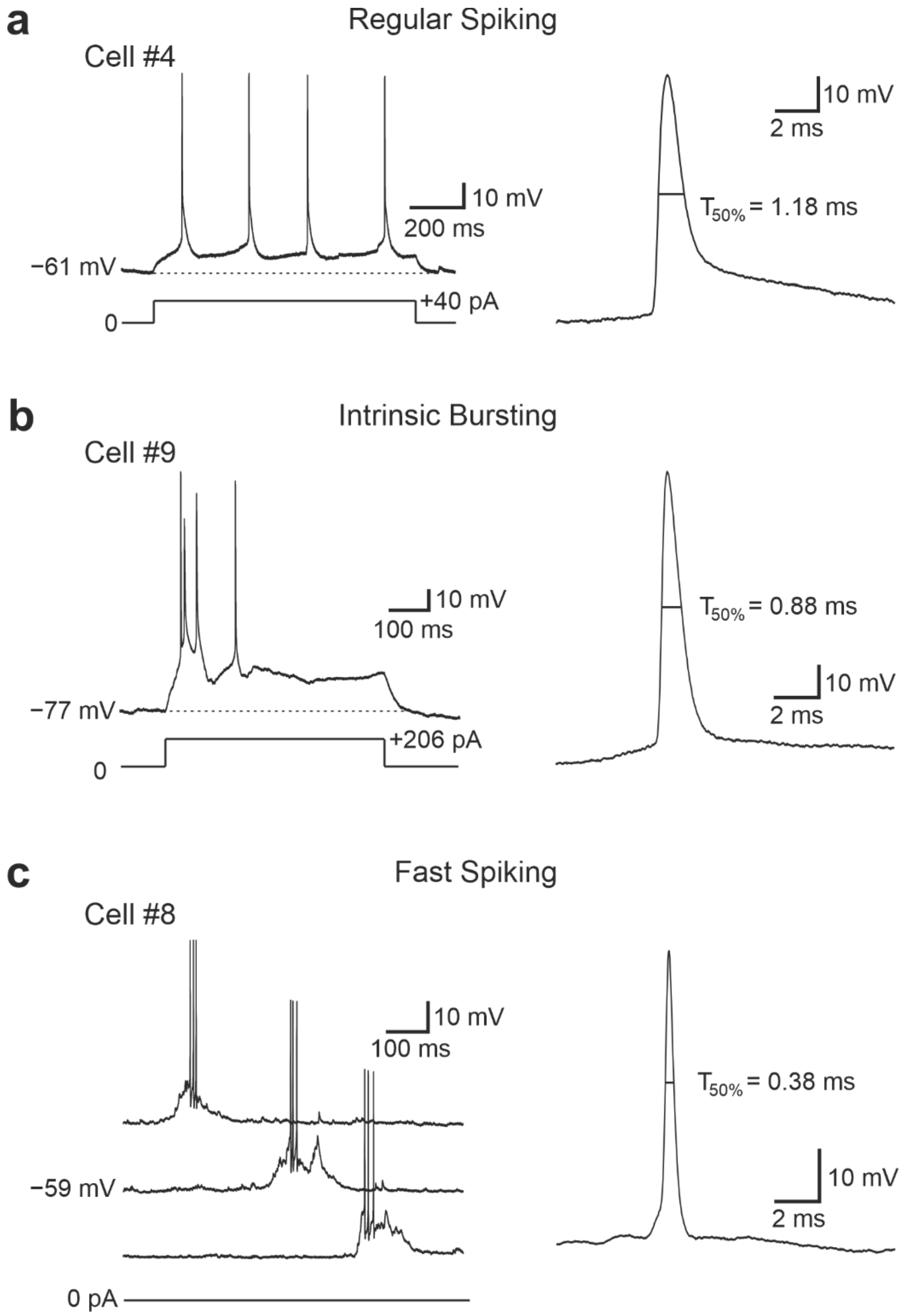

Supplementary Figure 3. Electrophysiological phenotypes of regular spiking, intrinsic bursting and fast spiking neurons in L5 of somatosensory cortex. Whole-cell current-clamp recordings from three types of L5 neurons identified as **a:** regular spiking (RS), **b:** intrinsic bursting (IB) and **c:** fast-spiking (FS) neurons based on their action potential (AP) firing patterns and AP waveform [1,2]. Passive membrane properties and AP waveform and firing patterns of all recorded neurons are summarized in the Supplementary Table 1, and change in membrane potential and neuronal firing during SDs are shown in the Supplementary Table 2.

Cell #	Type	Depth, μm	E_m, mV	R_m, MΩ	1st ISI, ms	2nd ISI, ms	AP Halfwidt, ms
**1**	IB	1230	-71	229	6	165	1.55
**2**	RS	1320	-58	160	130	105	1.03
**3**	IB	1280	-67	189	28	87	0.95
**4**	RS	1360	-64	178	225	241	1.17
**5**	IB	1330	-65	152	17	54	1.47
**6**	RS	1420	-63	59	-	-	0.72
**7**	RS	1110	-69	-	-	-	1.40
**8**	FS	1240	-59	-	-	-	0.37
**9**	IB	1340	-76	74	9	49	0.87
**10**	RS	1200	-67	223	84	159	2.60

Supplementary Table 1. Electrophysiological properties of L5 neurons. IB, intrinsic bursting neurons (olive); RS, regular spiking neurons (aqua); FS, fast spiking neuron (orange); E_m, membrane potential; R_m, membrane resistance; ISI, interspike intervals (first and second) between APs evoked by depolarizing current injection.

Cell # Type Depth	SD #	SD stop depth, µm	Pre-SD E_m, mV	Maximal E_m during SD, mV	SD - pre-SD E_m, mV	Inactivation threshold, mV	Maximal firing during SD, APs/s	AP Burst duration during SD, s
**Cell #1 IB 1230 µm**	1	840	-61.6	-46.5	15.1	-	9	8.8
	2	740	-67.3	-52.4	14.9	-	13	10.2
	3	1040	-64.3	-39.7	24.6	-42.2	19	8.1
	4	740	-68.0	-51.8	16.2	-	16	15.0
	5	940	-63.7	-42.4	21.4	-	20	7.8
	6	840	-63.8	-46.4	17.3	-	19	10.4
	7	1040	-63.3	-38.2	25.1	-43.8	20	9.1
	8	840	-68.1	-53.6	14.5	-	19	20.1
	9	740	-69.2	-55.1	14.1	-	19	15.6
	10	1240	-67.7	-32.7	35.0	-46.8	20	7.4
	11	840	-69.8	-53.0	16.8	-	16	12.4
	12	1140	-69.0	-43.1	25.9	-47.9	19	7.9
**Cell #2 RS 1320 µm**	1	1100	-54.0	-31.6	22.4	-36.5	31	8.4
	2	1900	-53.4	-10.1	43.3	-34.6	38	5.8
	3	800	-49.8	-32.6	17.1	-32.6	26	22.5
**Cell #3 IB 1280 µm**	1	1050	-65.5	-42.0	23.5	-	19	23.1
	2	1350	-61.8	-34.6	27.2	-34.7	16	10.6
	3	650	-59.9	-55.2	4.7	-	3	-
	4	1350	-57.8	-20.5	37.3	-33.0	16	3.6
	5	750	-59.4	-55.1	4.3	-	0	-
	6	1350	-60.2	-6.6	53.5	-29.5	23	4.2
	7	950	-62.9	-47.7	15.2	-	2	-
	8	1050	-60.0	-35.2	24.8	-	21	5.5
	9	1850	-56.9	-2.9	54.0	-27.0	21	3.6
	10	950	-61.2	-40.4	20.8	-	20	15.1
	11	1850	-55.6	3.3	58.9	-28.1	22	3.6
	12	1050	-56.9	-26.6	30.3	-28.2	27	5.0
	13	1850	-54.9	0.7	55.6	-28.7	27	4.1
	14	1050	-53.6	-24.3	29.3	-27.6	29	4.9
**Cell #4 RS 1360 µm**	1	850	-63.0	-42.8	20.2	-43.2	14	16.2
	2	1050	-63.5	-39.5	24.0	-43.9	12	11.2
	3	1050	-63.1	-38.7	24.4	-43.6	13	7.0
**Cell #5 IB 1330 µm**	1	400	-69.2	-67.2	2.0	-	2	-
	2	700	-70.3	-67.5	2.8	-	4	-
	3	800	-72.4	-67.1	5.3	-	2	-
	4	700	-69.5	-64.1	5.5	-	1	-
	5	1000	-73.4	-61.1	12.3	-	3	-
	6	1100	-73.5	-42.1	31.4	-	3	-
	7	1000	-73.5	-50.5	23.0	-	1	-
**Cell #6 RS 1420 µm**	1	800	-66.8	-45.7	21.2	-	24	18.7
	2	700	-63.0	-56.8	6.2	-	0	-
	3	900	-61.0	-33.0	27.9	-33.7	47	5.1
	4	600	-63.4	-54.7	8.8	-	1	-
	5	900	-58.6	-31.9	26.7	-31.9	47	6.1
	6	600	-58.0	-49.2	8.8	-	1	-
	7	1100	-57.7	-34.7	22.9	-35.2	50	4.6
**Cell #7, RS 1110 µm**	1	1850	-62.7	-21.5	41.2	-34.9	9	6.2
**Cell #8, FS 1240 µm**	1	550	-58.7	-35.2	23.5	-39.4	9	4.4
	2	550	-57.6	-33.1	24.5	-40.2	6	5.2
**Cell #9, IB 1340 µm**	1	1950	-59.7	-0.1	59.6	-	4	-
**Cell #10, RS 1200 µm**	1	1950	-59.4	2.7	62.1	-29.6	13	2.7
	2	1150	-68.3	-30.5	37.8	-36.4	8	2.7

Supplementary Table 2. Changes in membrane potential and action potential firing in deep cortical cells recorded during high-potassium induced SD of different propagation depth from the rat somatosensory cortex. IB, intrinsic bursting neuron (olive); RS, regular spiking neuron (aqua); FS, fast spiking neuron (orange); E_m, membrane potential. Cell ID# as in the Supplementary Table 1.

REFERENCES

1 Connors, B. W. & Gutnick, M. J. Intrinsic firing patterns of diverse neocortical neurons. *Trends Neurosci* **13**, 99-104 (1990).

2 Jacob, V., Petreanu, L., Wright, N., Svoboda, K. & Fox, K. Regular spiking and intrinsic bursting pyramidal cells show orthogonal forms of experience-dependent plasticity in layer V of barrel cortex. *Neuron* **73**, 391-404 (2012).
