# Supplementary material for: Diversity of cortical activity changes beyond depression during Spreading Depolarizations
Source: Nat Commun. 2023 Nov 25;14:7729. doi: 10.1038/s41467-023-43509-3 (PMC10676372; doi:10.1038/s41467-023-43509-3)
Supplement: Supplementary file 3 — Reporting Summary [file 41467_2023_43509_MOESM3_ESM.pdf]

Corresponding author(s): Roustem Khazipov

Last updated by author(s): Nov 3, 2023

Reporting Summary

Nature Portfolio wishes to improve the reproducibility of the work that we publish. This form provides structure for consistency and transparency in reporting. For further information on Nature Portfolio policies, see our Editorial Policies and the Editorial Policy Checklist.

Statistics

For all statistical analyses, confirm that the following items are present in the figure legend, table legend, main text, or Methods section.

n/a	Confirmed	
☐	☒	The exact sample size (n) for each experimental group/condition, given as a discrete number and unit of measurement
☐	☒	A statement on whether measurements were taken from distinct samples or whether the same sample was measured repeatedly
☐	☒	The statistical test(s) used AND whether they are one- or two-sided<br>*Only common tests should be described solely by name; describe more complex techniques in the Methods section.*
☒	☐	A description of all covariates tested
☐	☒	A description of any assumptions or corrections, such as tests of normality and adjustment for multiple comparisons
☐	☒	A full description of the statistical parameters including central tendency (e.g. means) or other basic estimates (e.g. regression coefficient) AND variation (e.g. standard deviation) or associated estimates of uncertainty (e.g. confidence intervals)
☐	☒	For null hypothesis testing, the test statistic (e.g. F, t, r) with confidence intervals, effect sizes, degrees of freedom and P value noted<br>*Give P values as exact values whenever suitable.*
☒	☐	For Bayesian analysis, information on the choice of priors and Markov chain Monte Carlo settings
☒	☐	For hierarchical and complex designs, identification of the appropriate level for tests and full reporting of outcomes
☐	☒	Estimates of effect sizes (e.g. Cohen's d, Pearson's r), indicating how they were calculated

Our web collection on statistics for biologists contains articles on many of the points above.

Software and code

Policy information about availability of computer code

Data collection	Animal data: Cheetah 6.3.2 (Digital Lynx, Neuralynx, USA), Clampex 10.3 (Axopatch 200B, Digidata 1440A, Molecular Devices, USA).<br>Clinical data: ECoG recording with BrainVision Recorder (Brain Products, Munich, Germany) and Powerlab 16/SP (ADInstruments, New South Wales, Australia). Manual detection of events with LabChart-8 (ADInstruments).
Data analysis	Matlab 2018b, Python. Detailed codes for the data analysis available upon request. Codes used to detect and analyze Spreading Depolarizations are available at https://doi.org/10.5281/zenodo.10066378

For manuscripts utilizing custom algorithms or software that are central to the research but not yet described in published literature, software must be made available to editors and reviewers. We strongly encourage code deposition in a community repository (e.g. GitHub). See the Nature Portfolio guidelines for submitting code & software for further information.

Data

Policy information about availability of data

All manuscripts must include a data availability statement. This statement should provide the following information, where applicable:

- Accession codes, unique identifiers, or web links for publicly available datasets
- A description of any restrictions on data availability
- For clinical datasets or third party data, please ensure that the statement adheres to our policy

Animal data supporting the results of the study are available from the authors upon request. Examples of the data described in Figure 2b are available at https://doi.org/10.5281/zenodo.10067055. Clinical data: Electronic recording, processing, and storage of the data were approved by the data protection officer of the Charite — Universitatsmedizin Berlin (data protection votes from May 28th 2008 and May 5th 2014). The datasets analyzed during the current study are not publicly available because the patient's informed consent only permits the data analysis and publication by the investigators.

Research involving human participants, their data, or biological material

Policy information about studies with human participants or human data. See also policy information about sex, gender (identity/presentation), and sexual orientation and race, ethnicity and racism.

Reporting on sex and gender

A subpopulation of 31 patients from the DISCHARGE-1 study (PMID: 35411920) was- included in the present study. We report the biological sex of our patients. This is important because aneurysmal subarachnoid hemorrhage (aSAH) is a typical disease with a strong imbalance between women and men. In our study, both sexes are represented (21 women (67.7%), 10 men (32.3%)). This proportion of both sexes in aSAH is consistent with the literature. If the patient was awake and oriented upon arrival at the emergency department, they could self-report their sex. Otherwise, the patient's sex was provided by family members or a legal representative, or was determined by examination of biological characteristics by medical personnel on arrival at the emergency department. Unlike sex, determination of gender requires self-assessment by the patient. In the DISCHARGE-1 trial, more than 50% of patients were continuously comatose until the main follow-up examination approximately 14 days after the initial hemorrhage. This means that in the period relevant for the primary objective of DISCHARGE-1, no self-assessment was possible for more than 50% of the patients and, accordingly, no answer to question about gender. In addition, a severe aSAH is an often devastating condition. Although SAH accounts for only —3% of all strokes and —5% of deaths from stroke, the relative youth of the affected individuals means that it is responsible for a quarter of all stroke-related years of potential life lost before age 65. Correspondingly, SAH is the third most common etiology leading to brain death during critical care. Accordingly, in the DISCHARGE-1 trial, 25.9% of patients had died by the time of the second follow-up at 7 months. At least another 22.2% of patients had not reached a state after 7 months in which they could have given information about their gender identity. This is a typical outcome of severe aSAH, although aSAH patients usually have good age-average health by the time of aneurysm bleeding. All this means that even if the question about gender identity had been included in our questionnaire, valid statements about it would not have been possible for almost half of the patients.

Reporting on race, ethnicity, or other socially relevant groupings

None of the socially constructed or socially relevant categorization variables were used in our manuscript.

Population characteristics

A subpopulation of 31 patients from the DISCHARGE-1 study (PMID: 35411920) was included in the present study (mean age 57±11 years). Population characteristics of the DISCHARGE-1 study: Median age = 55 years (IQR: 47-63) / Female = 67.2%, male = 32.8% / World Federation of Neurosurgical Societies (WFNS) grade I —V: I = 23.9%, II = 18.3%, III = 7.2%, IV = 22.2%, V = 28.3% / Modified Fisher Score (MFS) grade 0 - 4: 0 = 0%, 1= 3.3%, 2 = 2.8%, 3 = 18.9%, 4 = 75%/ Rosen-Macdonald Score (RMS) grade 1 — 15: 1-3 = 15%, 4-6 = 42.8%, 7-9 = 37.8%, 10-12 = 4.4%, 13-15 = 0%/ Location of aneurysm responsible for aSAH: middle cerebral artery = 37.2%, anterior communicating artery = 34.4, posterior communicating artery = 13.9%, internal carotid artery = 6.1%, anterior cerebral artery = 3.3%, posterior inferior cerebellar artery = 2.2%, basilar artery = 1.7%, pericallosal artery = 1.1% / Aneurysm treatment: clip ligation = 89.4%, coiling = 10.6% / 7 months outcome using the extended Glasgow Outcome Score (eGOS) grade 1— 8: 1-2 = 27.8%, 3-4 = 28.4%, 5-6 = 16.7%, 7-8 = 27.2%.

Recruitment

The 31 patients of the present study represent a randomly selected third of the Berlin cohort in DISCHARGE-1 who had direct current (DC)/ alternating current (AC)-electrocorticography (ECoG) recordings and at least one spreading depolarization.
Inclusion criteria of DISCHARGE-1: Ruptured saccular aneurysm proven by CT-angiography (CTA) or digital subtraction angiography (DSA) / Female or male patients at age 18 years / WFNS grade I - V unless the clinical state suggests an unfavorable prognosis such as wide, non-reactive pupils for more than 1 hour / Onset of clinical symptoms of aSAH within the preceding 72 hours / Either surgical treatment of the aneurysm via craniotomy or, in coiled patients, burr hole trepanation for placement of a ventricular drain or oxygen sensor, which allows the simultaneous placement of a subdural electrode strip / Informed consent is obtained from the patient or a legal representative.
Exclusion criteria of DISCHARGE-1: aSAH due to other causes / Admission in a clinical state with unfavorable prognosis / Bleeding diathesis / Cytostatic therapy in patients with malignant disease / Pregnancy / Unavailability of the monitoring equipment or insufficient staff / Refusal of the patient or legal representative to participate in the study / ECoG recording time of less than 24 hours between the assessment of the tissue loss due to early focal brain injury and the assessment of the tissue loss due to delayed cerebral ischemia.

Ethics oversight

The recordings from patients were carried out in accordance with protocols approved by the Charite — Universitatsmedizin Berlin, corporate member of Freie Universitat Berlin, Humboldt Universitat zu Berlin, and Berlin Institute of Health, Berlin, Germany (Ethical Committee Ethikausschuss CBF am Campus Benjamin Franklin, Ethical vote # EA4/022/09).

Note that full information on the approval of the study protocol must also be provided in the manuscript.

Field-specific reporting

Please select the one below that is the best fit for your research. If you are not sure, read the appropriate sections before making your selection.

☒ Life sciences ☐ Behavioural & social sciences ☐ Ecological, evolutionary & environmental sciences

For a reference copy of the document with all sections, see nature.com/documents/nr-reporting-summary-flat.pdf

Life sciences study design

All studies must disclose on these points even when the disclosure is negative.

Sample size	Animal data: sample sizes for animals and neurons comply with the criteria established in the field, are similar to those reported in our previous publications (References 18, 19, 21, 25, 27, 29, 30, 51, 65, 75), sufficient to ensure the validity of the results, and based on the 3R principles.<br>Clinical data: Primary objective of the prospective trial DISCHARGE-1 was to calculate: (i) sensitivity; and (ii) specificity for a known cut-off value for the peak total spreading depolarization (SD)-induced depression duration of a recording day (PTDDD) during the delayed neuromonitoring period (PTDDDdelayed) that indicates delayed ischemic infarcts ipsilateral to the recording strip as assessed by serial neuroimaging; and (iii) to estimate a new cut-off value. For this purpose, 180 of 205 (87.8%) patients could be analyzed as previously reported (PMID: 35411920). Because formal power analysis can only be performed for the primary objective (see http://www.isrctn.com/ISRCTN05667702), secondary study results, such as the diversity of cortical activity changes beyond depression during spreading depolarizations in the present case, are reported with the caveat that formal power analysis could not be performed.
Data exclusions	Animals: All animals and neurons with sufficient recording quality were included in this study.<br>Clinical: The 31 patients of the present study represent a randomly selected third of the Berlin cohort in DISCHARGE-1 who had direct current (DC)/ alternating current (AC)-electrocorticography (ECoG) recordings and at least one spreading depolarization. Data were excluded from analysis if presented only isoelectric SDs or poor recordings quality (n = 5 patients).
Replication	Results were consistent in each of 26 aSAH patients, 22 rats and 10 neurons analyzed independently.
Randomization	The clinical study is an observational study. No experiments were performed in the patients. From a larger cohort, we randomly selected patients for this substudy of DISCHARGE-1 using the sample() method from the Python random library.<br>Animal data set is from a single group of randomly selected animals. No more than 2 animals were from the same litter.
Blinding	Blinding was not relevant to the data collection, as the study was a single-group study in both human patients and animals.

Reporting for specific materials, systems and methods

We require information from authors about some types of materials, experimental systems and methods used in many studies. Here, indicate whether each material, system or method listed is relevant to your study. If you are not sure if a list item applies to your research, read the appropriate section before selecting a response.

Materials & experimental systems

n/a	Involved in the study	
☒	☐	Antibodies
☒	☐	Eukaryotic cell lines
☒	☐	Palaeontology and archaeology
☐	☒	Animals and other organisms
☐	☒	Clinical data
☒	☐	Dual use research of concern
☒	☐	Plants

Methods

n/a	Involved in the study	
☒	☐	ChIP-seq
☒	☐	Flow cytometry
☒	☐	MRI-based neuroimaging

Animals and other research organisms

Policy information about studies involving animals; ARRIVE guidelines recommended for reporting animal research, and Sex and Gender in Research

Laboratory animals	Wistar rats aged from 3 to 8 weeks were used.
Wild animals	No wild animals were used.
Reporting on sex	To be consistent with the human data, rats of both sexes were used in the study.

Field-collected samples	The study did not require field-collected samples.
Ethics oversight	Animal care and procedures were in accordance with EU Directive 2010/63/ EU for animal experiments, and all animal-use protocols were approved by the Local Ethical Committee of Kazan Federal University (#24/ 22.09.2020).

Note that full information on the approval of the study protocol must also be provided in the manuscript.

Clinical data

Policy information about clinical studies

All manuscripts should comply with the ICMJE guidelines for publication of clinical research and a completed CONSORT checklist must be included with all submissions.

Clinical trial registration	DISCHARGE-1 was pre-registered (http://www.isrctn.com/ISRCTN05667702).
Study protocol	The study protocol of DISCHARGE-1 is explained in great detail in the Supplementary Material of the publication (PMID: 35411920).
Data collection	The data collection of DISCHARGE-1 is explained in great detail in the Supplementary Material of the publication (PMID: 35411920).
Outcomes	Our study was an exploratory study. In the world-wide unique clinical dataset of DISCHARGE-1, it was our aim to study in detail the relationship of possible brain activity changes with spreading depolarization.

Plants

Seed stocks	*Report on the source of all seed stocks or other plant material used. If applicable, state the seed stock centre and catalogue number. If plant specimens were collected from the field, describe the collection location, date and sampling procedures.*
Novel plant genotypes	*Describe the methods by which all novel plant genotypes were produced. This includes those generated by transgenic approaches, gene editing, chemical/radiation-based mutagenesis and hybridization. For transgenic lines, describe the transformation method, the number of independent lines analyzed and the generation upon which experiments were performed. For gene-edited lines, describe the editor used, the endogenous sequence targeted for editing, the targeting guide RNA sequence (if applicable) and how the editor was applied.*
Authentication	*Describe any authentication procedures for each seed stock used or novel genotype generated. Describe any experiments used to assess the effect of a mutation and, where applicable, how potential secondary effects (e.g. second site T-DNA insertions, mosiacism, off-target gene editing) were examined.*
